# Reducing Functional Domain of Histatin 5 Improves Antifungal Activity and Prevents Proteolytic Degradation

**DOI:** 10.3390/microorganisms13051091

**Published:** 2025-05-08

**Authors:** Carolina R. Zambom, Gabriel Bernardes, Fauller Henrique da Fonseca, Gabriela Vieira Silva Zolin, Mariana de Melo Faceto Portella, Lina Maria Marin, Edson Crusca, Ed S. Krol, Walter L. Siqueira, Saulo Santesso Garrido

**Affiliations:** 1 Institute of Chemistry, Department of Biochemistry and Organic Chemistry, UNESP—Sao Paulo State University, Araraquara 14800-060, Brazil; fauller.henrique@unesp.br (F.H.d.F.); gabriela.zolin@unesp.br (G.V.S.Z.); mariana.portella@unesp.br (M.d.M.F.P.); ecrusca@gmail.com (E.C.); 2 College of Pharmacy and Nutrition, University of Saskatchewan, Saskatoon, SK S7N 5E5, Canada; gabriel.dalio@usask.ca (G.B.); ed.krol@usask.ca (E.S.K.); 3 College of Dentistry, University of Saskatchewan, Saskatoon, SK S7N 5E4, Canada; lina.marin@usask.ca (L.M.M.); walter.siqueira@usask.ca (W.L.S.)

**Keywords:** histatin 5, P-113, proteolysis, degradation, saliva, peptides, SPPS, antifungal peptides

## Abstract

Histatin 5 (Hst5) is an antifungal peptide (AFP) naturally produced by parotid glands with strong activity against *Candida albicans*. One of its mechanisms of action is the generation of reactive oxygen species (ROS) inside the *C. albicans* cells. Despite being an important peptide for the human innate immune response, its activity is reduced or inactivated by proteolytic degradation caused by salivary enzymes. To overcome this barrier, we used solid phase peptide synthesis (SPPS) to modify the Hst5 amino acid sequence improving its antifungal action and minimizing its degradation. We synthesized five peptides, three of which were based on the Hst5 functional domain. We determined that the smallest peptides (8WH5, 7WH5 and 6WH5) demonstrated the greatest antifungal action against *C. albicans*, including one fluconazole-resistant strain. Besides that, cationic-PAGE and HPLC assays showed that the degradation in saliva was slower for the smaller peptides than for 0WHst5 and WP113. Furthermore, 8WH5, 7WH5 and 6WH5 were found in the samples even after 8 h in whole saliva, while 0WHst5 and WP113 completely disappear after 1.5 h. Finally, we found that the smaller peptides were less fragmented than the 0WHst5 and WP113, so they were the smallest fragments of Hst5 to preserve its antifungal action with reduced degradation in whole saliva. Thus, they can be considered promising molecules for the treatment of *C. albicans* in the oral cavity.

## 1. Introduction

Antimicrobial peptides (AMPs), including antifungal peptides (AFP), are an important class of molecules that are naturally found in almost all living organisms. The AMPs provide a first line defense against pathogens, contributing to the prevention of infections by fungi, bacteria, viruses, protozoa, and other parasites. Several AMPs are part of the human innate immune system and continuously protect the human body, suggesting therapeutic potential as antibiotics [1,2]. Unlike some conventional antibiotics, it is more difficult to acquire resistance against these peptides, as they have more than one mechanism of action. For this reason, a single peptide could act on different metabolic pathways and different organelles to cause death. There are many potential advantages in the use of AMPs, such as their broad spectrum and the low concentration required for activity without severe side effects [3]. Due to their great importance and diversity, AMPs are gaining momentum in the fight against antimicrobial resistance, including inhibition of multi-drug-resistant pathogens [2,4,5,6]. In this context, peptides like α and β defensins, cathelicidins and histatins are among the AFPs with widen antimicrobial action [7].

Naturally found in human saliva, the histatins are a class of histidine-rich peptides that possess antimicrobial activity, in addition to playing an important role in wound healing and oral re-epithelialization. Histatin-5 (Hst5) is the peptide with the greatest antifungal action of the histatin family and has activity against some pathogenic microorganisms, including *Candida albicans* [8]. High levels of Hst5 were found in the saliva of patients with periodontal and oral fungal infections. This demonstrates the physiological response of the human body against these infections [9,10,11]. Furthermore, a minimum inhibitory concentration (MIC) of Hst5 has been reported in the range of 8–16 μg mL^−1^ for some species of *C. albicans* [12,13]. It has also been shown that Hst5 is capable of inhibiting strains of *Candida auris* and other multi-drug-resistant pathogens such as *Enterococcus faecium*, *Staphylococcus aureus*, *Klebsiella pneumoniae*, *Acinetobacter baumanii* and *Pseudomonas aeruginosa* [14,15].

Because of its relevance for the maintenance of oral homeostasis and its role in combating oral infections, Hst5 is a widely studied peptide. Its mechanism of action involves the release of ATP to the extracellular environment through interaction of Hst5 with the microorganism’s mitochondria [16,17]. This lack of energy causes death, but some studies have shown that in addition to causing ATP efflux, Hst5 also generates reactive oxygen species (ROS) inside the cell and interferes with the G1 phase of the cell cycle. However, Hst5 activity may be reduced by proteolytic cleavage mediated by enzymes present in saliva or by *C. albicans*, which has cleavage action at specific sites that it can recognize along the peptide structure. Besides that, some studies found that a minimum of 12 amino acid residues at the C terminus (functional domain) were required for the killing of fungal cells [18]. P-113, a 12-amino acid fragment of Hst5, was synthesized and tested against some Candida strains, demonstrating potent activity against *Candida albicans*, *Candida glabrata*, *Candida parapsilosis* and *Candida tropicalis* [19]. In addition, some modifications in the amino acid sequence of this peptide can promote oral mucosa repair, inhibit periodontal pathogens, and control the dental biofilms formation [20,21].

However, P-113 is not the only Hst5-derived fragment ever synthesized. Over the years, several other peptides have been produced based on the Hst5 amino acid sequence with the intention to improve its antifungal action [8,15,22,23,24,25,26,27]. Changes in the amino acid sequence and production of hybrid or chimeric peptides are some of the modifications that have been made in the Hst5 sequence to increase its antifungal activity. Also, the production of analogues based on Hst5 can overcome other problems associated with this peptide, like its rapid degradation by proteolytic enzymes present in human saliva [28,29,30]. For this reason, Hst5 antifungal action is reduced or inactivated and its use to treat fungal infections in the oral cavity becomes a challenge.

Therefore, it is important to propose strategies to increase Hst5 antifungal action and prevent the proteolytic degradation caused by salivary enzymes. The solid phase peptide synthesis (SPPS) pioneered in 1963 by Merrifield is an important tool for the creation and modification of bioactive peptides such as Hst5 [31]. Nowadays, the SPPS strategy is continuously progressing, making it possible to modify the peptide amino acid chain by decreasing or increasing the number of amino acids [32]. In this study, using SPPS, we synthesized five peptides to compare their antifungal activities. The already known peptides Hst5 and P-113 were synthesized here with a minor modification and, based on the Hst5 12-amino acid sequence known as the functional domain, we prepared three novel small peptides. We hypothesize that the small peptides could be more resistant to proteolytic cleavage with improved antifungal activity.

## 2. Materials and Methods

### 2.1. Peptide Synthesis

The peptides were synthesized manually as described previously by Zambom et al. [33]. Briefly, in each synthetic cycle, the α-amino group deprotection was performed with 20% piperidine in dimethylformamide (DMF) (Sigma-Aldrich, São Paulo, Brazil) for 20 min. The coupling reactions were performed with a threefold excess of diisopropyl-carbodiimide (DIC) (Sigma-Aldrich, São Paulo, Brazil) and N-hydroxybenzotriazole (HoBt) (Sigma-Aldrich, São Paulo, Brazil) in DMF/methylene chloride (DCM) (1:1, *v*:*v*). After approximately 2 h of coupling, the ninhydrin (Sigma-Aldrich, São Paulo, Brazil) test was performed to confirm the occurrence of the reaction. Final cleavage of the peptides from the resins and the deprotection of the side-chain protector groups were performed using a *v*/*v* solution containing TFA (94.5%) (Sigma-Aldrich, São Paulo, Brazil), deionized water (2.5%), EDT (2.5%) (Sigma-Aldrich, São Paulo, Brazil) and TIS (0.5%) (Sigma-Aldrich, São Paulo, Brazil) at 25 °C for 3 h. After the cleavage procedure, the crude peptides were precipitated with ethyl ether, separated from the soluble non-peptidic contents by centrifugation, extracted into 10% acetic acid in water and lyophilized.

The crude peptides were dissolved in solvent A (0.045% TFA.H_2_O) and purified by semi-preparative HPLC with a Zorbax Eclipse XDB reverse phase C18 column (9.4 mm × 250 mm and 5 µm) (Agilent, São Paulo, Brazil). The qualitative analysis was performed analytically using a Shimadzu LC-10A/C-47A separation system coupled to a Shimadzu LC-10A/C-47A UV/Vis detector (Shimadzu, São Paulo, Brazil) with a Waters Symmetry C18 column (2.1 mm × 150 mm and 5 μm) (Waters, Barueri, Brazil). The chromatographic conditions in the semi-preparative mode were: solvents A (0.045% *v*/*v* TFA/H_2_O) and B (0.036% *v*/*v* TFA/ACN), gradient of 0.33%/min solvent B for 90 min, flow rate of 5 mL min^−1^ and detection wavelength at 220 nm. For the analytical mode, the conditions for solvents A (0.045% TFA/H_2_O) and B (0.036% TFA/ACN) were a gradient from 5 to 95% solvent B for 30 min, flow rate of 0.6 mL min^−1^ and detection wavelength at 220 nm. After the purification procedure, the peptide characterization was carried out using mass spectrometry. The analysis of pure peptides was performed by HPLC coupled to a Bruker^®^ Ion Trap Amazon SL mass spectrometer (Bruker, São Paulo, Brazil) operated in electrospray positive mode (LC/ESI-MS^+^).

### 2.2. Microorganisms and Growing Conditions

For the antifungal tests, the strains *C. albicans* ATCC 90028, *C. albicans* ATCC 18804 and *C. albicans* ATCC 10231 were donated by the National Institute of Quality Control in Health (INCQS—Fundação Oswaldo Cruz—Fiocruz, Rio de Janeiro, Brazil). To prepare *C. albicans* for the tests, the lyophilized microorganism was initially reactivated in Sabouraud Dextrose Broth (Kasvi, Pinhais, Brazil) and incubated at 37 °C for 24 h. Then, the yeasts were seeded in Sabouraud Dextrose Agar and incubated for 48 h at 37 °C. Finally, the stock culture obtained was stored in a refrigerator at 4 °C for 3 months.

To prepare the standardized suspensions, some colonies of *C. albicans* stock cultures were seeded in Sabouraud Dextrose Broth and incubated for 24 h at 37 °C. After this time, a small aliquot of the inoculum was transferred to sterile Sabouraud Dextrose Broth to obtain a suspension with optical density (OD) between 0.10 and 0.15 (530 nm), with approximate concentration of 1 × 10^6^ CFU mL^−1^ [34,35]. Finally, the suspension was diluted to obtain a concentration of 1 × 10^3^ CFU mL^−1^, which was used in the tests described below.

### 2.3. Antifungal Assay

The antifungal activity tests were performed using broth microdilution. The broth microdilution was performed as described in the M27-A3 document of Clinical and Laboratory Standards Institute (CLSI) with modifications [36]. The medium used was Sabouraud Dextrose Broth (SDB). The synthetic peptides were dissolved in a Tris·HCl 10 mM pH 7.2 buffer and added to the plate containing SDB for a final concentration in the first well of 160 µmol L^−1^. The cell suspension was prepared as described under “Microorganisms and growth conditions”. This suspension was inoculated on a microdilution plate previously prepared with the peptides diluted in a concentration range from 160 to 0.31 µmol L^−1^. The control drug used was fluconazole (FLU), diluted in deionized water. The plates were incubated at 37 °C for 48 h and at the end of the incubation time, the OD in each well was read in a spectrophotometer at 595 nm. The test was performed in triplicate for each of the strains.

### 2.4. C. albicans Cell Viability

*C. albicans* ATCC 90028 was used for this assay. The initial microorganism solution was performed as described under “Microorganisms and growth conditions” with some modifications. To prepare the microorganism solution, some colonies of *C. albicans* from stock cultures were seeded in Sabouraud Dextrose Broth and incubated for 24 h at 37 °C. After this time, an aliquot of the microorganism suspension was transferred to sterile Sabouraud Dextrose broth in order to obtain a suspension of microorganisms with optical density (OD) of 0.38 (520 nm), which indicates a concentration of 1 × 10^7^ CFU mL ^−1^ [37,38].

In a microplate, serial dilutions of 200 µmol L^−1^ to 6.25 µmol L^−1^ of the peptides 0WHst5, WP113, 8WH5, 7WH5 and 6WH5, diluted in Tris-HCl 10 mM pH 7.2 buffer, were performed. Afterwards, 50 µL of the previously prepared *C. albicans* suspension was added to each well containing 50 µL of each diluted peptide. For control group, 50 µL of *C. albicans* was added to 50 µL of saline solution (0.85% NaCl). Then, the plate was incubated for 2 h at 37 °C. After the incubation time, 50 µL of suspension from the selected wells was diluted in 9 mL of saline solution. After that, 25 µL aliquots of the diluted suspension were plated on SDA and incubated for 48 h at 37 °C. Colony counting was used to assess cell viability (CFU mL^−1^). This experiment was carried out in triplicate; data are present as the mean ± SD. The results were evaluated statistically by ANOVA and Tukey post hoc test was used to determine differences between means (a = 0.05). Peptides were statistically compared between treatments of the same concentration. Equal letters indicate statistically equal means while different letters indicate statistically different means.

### 2.5. Saliva Collection

All methods described here were carried out in accordance with relevant guidelines and regulations. All subjects gave their informed consent for inclusion before they participated in the study. The study was conducted in accordance with the Declaration of Helsinki, and the protocol was approved by the Research Human Ethics Board of the University of Saskatchewan (review number #1597). After an informed consent was obtained from all participants, stimulated whole saliva (WS) samples were collected from healthy, non-smoking adult male and female volunteers ranging from 25 to 39 years old. WS was obtained for three volunteers with good general oral health who were not taking medications that could alter their salivary flow and was performed as described previously in [29,39]. Salivary secretion was stimulated by chewing a piece of Parafilm wax. WS was collected between 9 and 11 am, at least 2 h after the last meal. The salivary secretion was collected using a collecting tube that was kept on ice until obtaining 5.0 mL. The total volume of collected saliva was centrifuged at 4500× *g* (Sorvall™ ST 16 Centrifuge—Thermo Fisher Scientific, Waltham, MA, USA) at 4 °C for a period of 30 min to separate bacteria, cells and residues. The total whole saliva pellet and the total whole saliva supernatant (WSS) were separated, and the WSS was pooled and used for the degradation assay.

### 2.6. Degradation of Synthetized Peptides in WSS

To assess and compare proteolysis degradation in the WS environment, all the peptides were added to WSS to observe their degradation over time by cationic-PAGE and HPLC. To investigate the degradation of each peptide, 50 µg mL^−1^ of synthetic peptides 0WHst5, WP113, 8WH5, 7WH5 and 6WH5 were added to 10-fold diluted WSS. After 0, 0.5, 1.5, 4, 6, 8, 24 and 48 h of incubation, two aliquots of 100 µL were removed (one for the cationic-PAGE analysis another for HPLC analysis). After that, the aliquots were boiled for 5 min to terminate proteolytic activity. Subsequently the aliquots were diluted in a buffer containing 80% acetonitrile/19.9% H_2_O/0.1% trifluoroacetic acid, dried using a Vacufuge concentrator (Eppendorf, Westbury, NY, USA) and desalted using ZipTip^®^ Pipette Tips (Merk, Rahway, NJ, USA) to concentrate and purify the samples [29].

#### 2.6.1. Cationic-PAGE

For the cationic-PAGE, the aliquots were dried using a Vacufuge concentrator and resuspended in sample buffer containing 0.04% methyl green (Thermo Fisher Scientific, Waltham, MA, USA) in 40% sucrose. Then, 5 μg of each peptide was used as standard for the test. The stacking and separation gels were polymerized using 100 W incandescent light for 9 min each and the electrophoresis was carried out at a constant voltage of 120 V. The gels were stained with 0.1% Coomassie brilliant blue (*w*/*v*), 8% methanol (*v*/*v*) and 7% acetic acid (*v*/*v*) overnight with agitation. Destaining was carried out with 40% methanol, 10% acetic acid and 50% water (*v*/*v*). The extent of degradation was quantified by Image Lab using the relative quantity tool.

#### 2.6.2. HPLC

The aliquots collected for the HPLC analysis were also dried, desalted and resuspended in 30 μL of 10 mM Tris·HCl pH 7.2 buffer solution. Then, 30 µL was analyzed by HPLC in analytical mode using a Waters Symmetry^®^ C18 5 µm, 4.6 × 250 mm reverse phase column (Part No. WAT054275) (Waters, Milford, MA, USA). The solvents used were all chromatographic grade (purchase from Sigma-Aldrich^®^, St. Louis, MO, USA) and the water used was ultrapure. The chromatographic conditions were solvents A (0.045% TFA/H_2_O) and B (0.036% TFA/ACN), in a gradient of 5% to 95% B in 30 min, with a flow of 0.53 mL min^−1^ at a detector wavelength of 280 nm, using a Waters Alliance 2695 HPLC connected to a Waters 2996 photodiode array detector (Waters, Milford, MA, USA).

#### 2.6.3. LC-ESI-MS/MS

After cationic-PAGE analysis, the bands were removed from the gels and underwent treatment for destaining and removal of the possible peptide fragments present. After that, dried samples were resuspended in 10 µL of 97.5% H_2_O/2.4% acetonitrile/1% formic acid, and were subsequently subjected to reverse-phase LC-ESI-MS/MS. The tandem mass spectrometry analysis was performed according to the pervious protocol Briefly, a Velos LTQ (Thermo-Finnigan, San Jose, CA, USA) was used, which allows liquid chromatography to be performed in line with a capillary column C18 connected to the mass spectrometer using electrospray ionization on a test scanner in the m/z values range of 390–2000 and at the same time performing tandem MS/MS analysis. The 50 μm × 10 cm reverse phase HPLC capillary column was packed in the laboratory using a Magic C18 resin 3 μm diameter and 200 Å pore size (Michrom BioResources, Auburn, CA, USA). The column was developed with a linear gradient of solvent B (acetonitrile containing 0.1% formic acid) from 8% to 50% in 60 min, and 50% to 100% in 25 min at a flow rate of 200 nL/minute. The electrospray voltage and the ion transfer capillary temperature were 1.8 kV and 250 °C, respectively.

#### 2.6.4. Data Analysis

The obtained MS/MS spectra were searched against human protein databases (Swiss Prot and TrEMBL, Swiss Institute of Bioinformatics, Geneva, Switzerland, http://ca.expasy.org/sprot/, accessed on 12 March 2024) using SEQUEST algorithm in Proteome Discoverer 2.4 software (Thermo Scientific, San Jose, CA, USA). Search results were filtered for a false discovery rate of 1% employing a decoy search strategy utilizing a reverse database.

## 3. Results and Discussion

### 3.1. Peptide Design

We have designed three new peptides based on the Hst5 amino acid sequence. Previous studies have shown that some amino acids present in the sequence of this peptide are preferentially attacked by salivary proteases. In addition, other studies suggest that the functional domain of the Hst5 is the 12-amino acid sequence known as P113 (“A K R H H G Y K R K F H”). Based on this fact, for the development of the peptides presented in this work, it was decided to maintain most of the functional domain of Hst5 and remove some amino acids that are targets of attacks by salivary proteases [19].

With the intention of exploring the antifungal activity of smaller fragments derived from Hst5, the peptides 8W5H, 7WH5 and 6WH5 were engineered and synthesized. The sequences of the peptides are listed in Table 1. As can be seen, we started by removing amino acids from WP113. We removed a N-terminal Ala, and C-terminal Lys, Phe and His to produce an 8-amino-acid peptide (8WH5). We then removed an additional Arg for the 7-amino-acid peptide (7WH5). Finally, we removed one more Lys from the N-terminal side, yielding our 6-amino-acid peptide (6WH5). This allowed us to investigate the antifungal properties of these novel peptides and to determine their ability to resist proteolytic cleavage caused by enzymes present in human saliva.

All peptides were obtained with greater than 96% purity as determined by HPLC coupled to a Bruker^®^ Ion Trap Amazon SL mass spectrometer operated in electrospray positive mode (LC/ESI-MS^+^). We used the tryptophan amino acid as a probe to allow analysis by fluorescence emission spectroscopy for better quantification of the peptides. We made this modification to all peptides to ensure that possible stability differences were caused mainly by the truncation process. In this context, 0WHst5 is a peptide analogous to Hst5 with only the addition of the amino acid tryptophan (W).

### 3.2. Peptide Antifungal Activity

**Figure 1:**
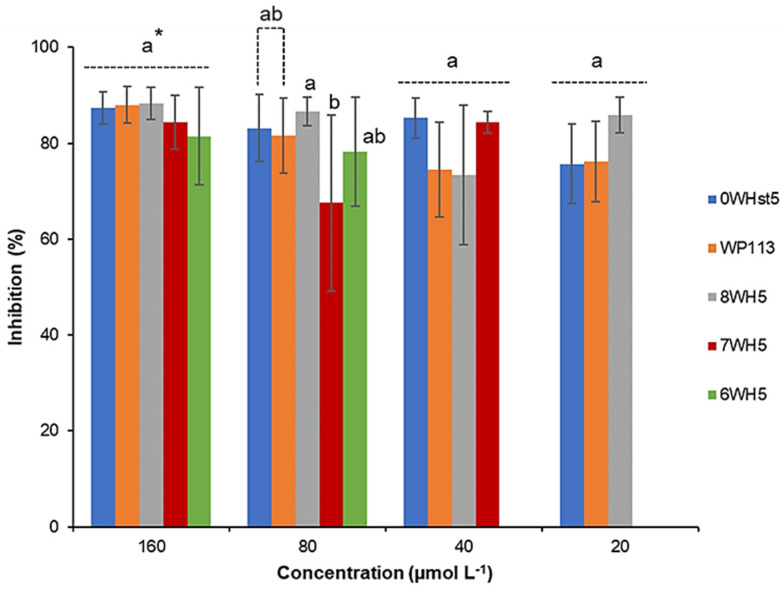
Inhibition of *C. albicans* ATCC 90028 by 0WHst5, WP113, 8WH5, 7WH5 and 6WH5. The experiment was performed in triplicate. * Peptides were statistically compared between treatments of the same concentration. Equal letters indicate statistically equal means while different letters indicate statistically different means.

Analysis of the antifungal activity of the peptides was carried out to evaluate and compare them with each other. To accomplish this, we used three strains of *C. albicans* (ATCC 10231, ATCC 18804 and ATCC 90028). ATCC 10231 is resistant to fluconazole, the most frequently used drug in treatments against *C. albicans*. As can be seen in Figure 1, for ATCC 90028, 20 µmol L^−1^ of 0WHst5 was able to inhibit 75% of the microorganism, which represents the same inhibition range that was described by Moffa et al. [13]. For the same strain, 20 µmol L^−1^ of the WP113 was able to inhibit 76% of the microorganism. No statistical difference was found between 0WHst5 and WP113 at 20 µmol L^−1^ for ATCC 90028. There are reports that describe a greater antifungal activity for WP113 when compared to 0WHst5 [19]; some of these studies use different *C. albicans* strains from the ones used here, which may explain the different results [12,19].

**Figure 2:**
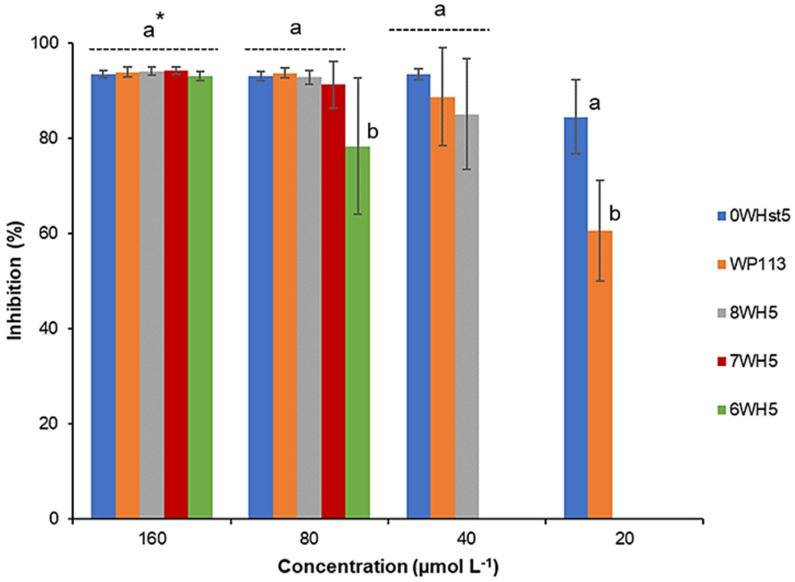
Inhibition of *C. albicans* ATCC 18801 by 0WHst5, WP113, 8WH5, 7WH5 and 6WH5. The experiment was performed in triplicate. * Peptides were statistically compared between treatments of the same concentration. Equal letters indicate statistically equal means while different letters indicate statistically different means.

For ATCC 18804 (Figure 2), greater than 80% inhibition was attained when 20 µmol L^−1^ of the 0WHst5 was used. We observed inhibition above 80% for 8WH5 (40 µmol L^−1^), 7WH5 (80 µmol L^−1^) and 6WH5 (160 µmol L^−1^). It is important to note that at 80 µmol L^−1^, 6WH5 presented inhibition above 70%. Even though this inhibition is statistically different and smaller than the others, this is a result that reveals that this small fragment derived from Hst5 still maintains its antifungal activity.

**Figure 3:**
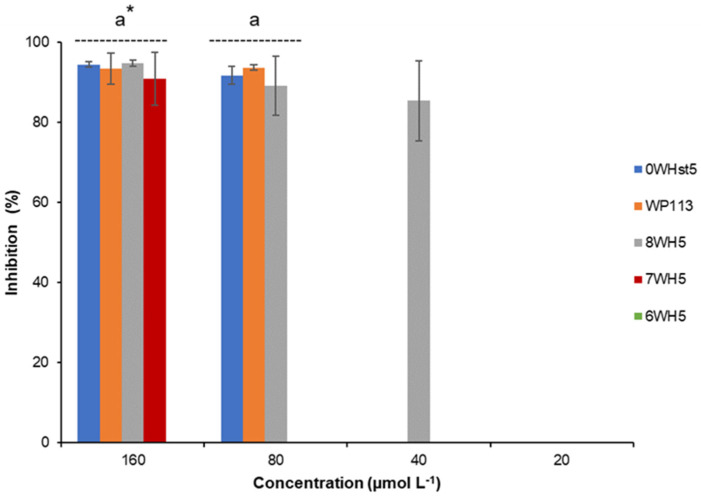
Inhibition of *C. albicans* ATCC 10231 by 0WHst5, WP113, 8WH5, 7WH5 and 6WH5. The experiment was performed in triplicate. * Peptides were statistically compared between treatments of the same concentration. Equal letters indicate statistically equal means while different letters indicate statistically different means.

For ATCC 10231 (Figure 3), only the 8-amino-acid peptide (8WH5) was able to inhibit 85% of growth at a concentration of 40 µmol L^−1^. However, at concentrations of 80 µmol L^−1^ the peptides 0WHst5 and WP113 showed antifungal activity against this fluconazole-resistant strain. 7WH5 also showed inhibition of 90.9% for this strain at a concentration of 160 µmol L^−1^. This result supports the use of SPPS to produce new peptides derived from known molecules, in this case Hst5, with the potential to be an important tool to overcome barriers to drug resistance. As we have demonstrated, these three novel peptides inhibited *C. albicans*, including drug-resistant strains.

Although previous reports have already described the antifungal activity of the P113, the same results could be observed for WP113 because their amino acid sequences differ only by the addition of tryptophan in the amino terminal region [12,19]. The other peptides are not yet described in the literature as antifungals. 8WH5 is described as a degradation product of Hst5 present in human saliva, but its antifungal potential had not yet been investigated [28]. As can be seen, peptides 7WH5 and 6WH5 also showed *C. albicans* inhibition. The inhibition potential of these peptides was less than 8WH5 or WP113, but it is interesting to note that even for the fluconazole resistant strain (Figure 3) the 7WH5 peptide showed inhibition at 160 µmol L^−1^. Thus, the results obtained demonstrated that smaller sequences could still retain antimicrobial activity. This strategy can allow the future clinical application of these peptides, once it makes the synthesis process faster.

### 3.3. Colony Counting Viability

**Figure 4:**
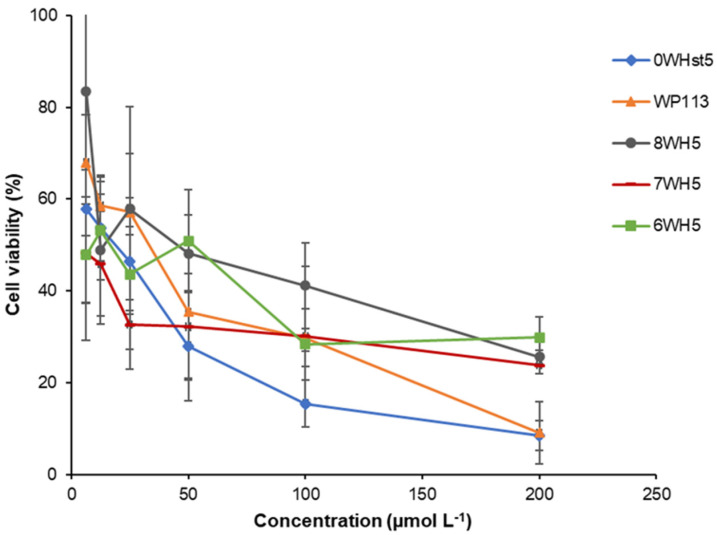
Cell viability of *C. albicans* ATCC 90028 cells after 2 h incubation at 37 °C with dilution series of peptides 0WHst5, WP113, W8H5, 7WH5 and 6WH5. The dilutions were then plated in Sabouraud Dextrose Agar (SDA) media and the logarithm values of CFU mL^−1^ were calculated for the determination of cell viability.

The *C. albicans* viability assay was performed to confirm the antimicrobial activity of the peptides. In this test, the results were obtained through colony counting (CFU mL^−1^) and then the values were converted into percentage of cell viability as represented in the graph in Figure 4. This graph shows the effect of the decrease in cell viability with the increase in peptide concentration.

After incubation for only 2 h, it could be seen that for 0WHst5, at a concentration of 200 µmol L^−1^, there was a reduction of viable *C. albicans* cells to just 10%. This result agrees with Moffa et al., who demonstrated that there was no statistical difference in growth inhibition between treatments in the range of 800 µg mL^−1^ to 25 µg mL^−1^ [13].

Therefore, at 200 µmol L^−1^, there were no statistical differences (a = 0.05) for the original peptide (0WHst5) and its structural analogue (WP113), with a reduced cell viability to 10%. 8WH5, 7WH5 and 6WH5 showed a reduction in the viable cells to approximately 30% at the concentrations of 100 and 200 µmol L^−1^, with no statistical difference between them. However, at 200 µmol L^−1^, the effects of peptides 0WHst5 and PW113 were statistically different from those of 8WH5, 7WH5 and 6WH5. Furthermore, the concentration of lower cell viability was 200 µmol L^−1^ for all peptides tested. It is possible to relate the results found in the cell viability assay (Figure 4) with the results found in the antifungal assay for ATCC 90028 (Figure 1). Thus, concentrations above 160 µmol L^−1^ inhibited 90% of *C. albicans* growth, resulting in only 10% cell viability.

Our results confirm that all synthesized peptides have antifungal action and show cell viability below 30% at a concentration of 100 µmol L^−1^.

### 3.4. Degradation of 0WHst5, WP113, 8WH5, 7WH5 and 6WH5 in WSS

**Figure 5:**
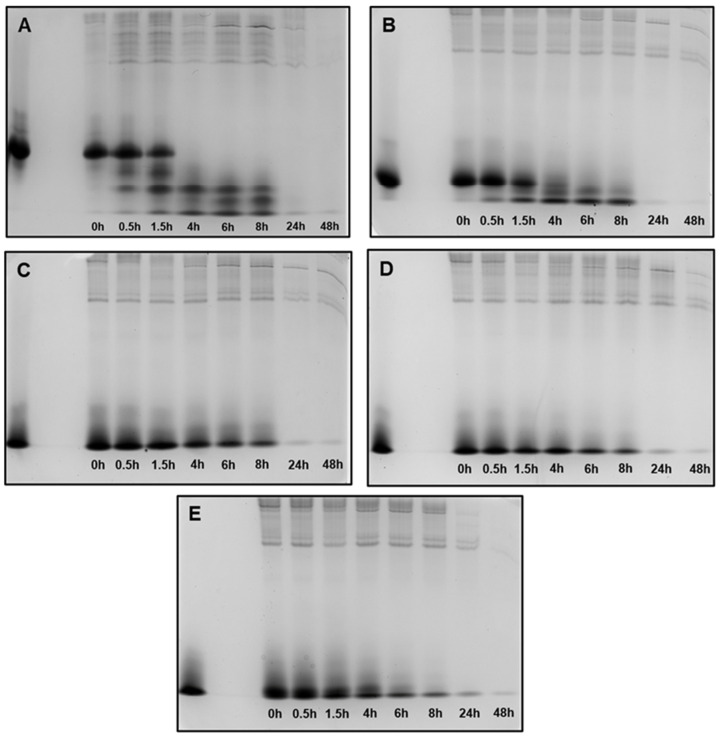
Peptide degradation profile in human saliva (WSS): (**A**) 0WHst5; (**B**) WP113; (**C**) 8WH5; (**D**) 7WH5; (**E**) 6WH5. An aliquot of 100 µL was collected after each incubation period and evaluated by cationic-PAGE. The first column on the left (column 1) corresponds to the standard band for all peptides tested; columns 2 to 9 show the different incubation periods (t = 0, 0.5, 1.5, 4, 6, 8, 24 and 48 h).

Human whole saliva is a biological fluid with high proteolytic activity [40,41,42]. This fluid has a complex composition with secretions from salivary glands, crevicular gingival fluid, oral epithelium and even microorganisms residing in the oral cavity [29,43,44]. For this reason, human whole saliva presents several types of proteases that can interfere with and modify salivary proteins or peptides [29]. In this work, we incubated all the synthesized peptides in diluted whole saliva supernatant (WSS) to mimic its proteolytic action in vitro. The aliquots obtained after degradation assay were assessed on a cationic polyacrylamide gel (cationic-PAGE), as shown in Figure 5. Previous studies have used this type of procedure for peptides of the histatin family, and specifically for Hst5 [28,29,39,45,46]. This type of gel facilitates the separation of cationic peptides such as histatins, and prevents the migration of negatively charged proteins that could mix with the positive peptides [29].

When in contact with diluted WSS, Hst5 is rapidly degraded: 60 min is the time reported for its complete degradation [29]. According to the images in Figure 5A, after 1.5 h of incubation, we were no longer able to observe the representative band for Hst5 in the gel. Similarly, Thomadaki et al. reported that 1.5 h was enough for the complete degradation of Hst5 [45]. After quantification of this band, we observed that 61% of 0WHst5 was still in the aliquot (Table 2). However, we observed total degradation in the next few hours as this band was no longer observed within 4 h, and quantification was not possible.

No reports were found in the literature about the degradation profile in human saliva for P113. Like P113, the synthetic WP113 conserves half of the original Hst5 chain [19], and like 0WHst5, WP113 also showed rapid degradation in diluted WSS. When observing the image in Figure 5B, we noted that the band referenced to this peptide was visible at 1.5 h and it was still possible to observe a weak band at 4 h. After quantification, 53.6% of this peptide was present in the 1.5-hour aliquot and only 7.23% in the 4-hour aliquot (Table 2). After that time, there was complete degradation.

**Figure 6:**
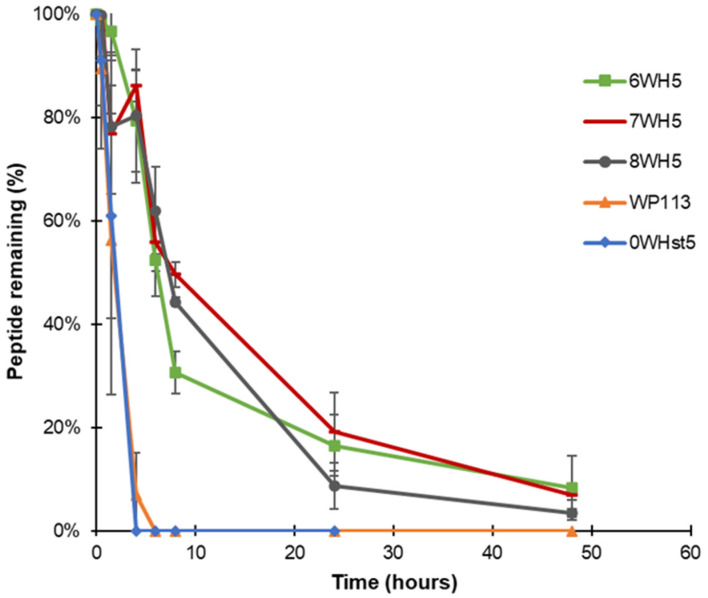
Peptide degradation over time for 0WHst5, WP113, 8WH5, 7WH5 and 6WH5. Data were obtained after the cationic-PAGE and the amount of peptide in each aliquot was determined using the software Image Lab 6.1 (Bio-Rad Inc.). Error bars correspond to the standard deviations on duplicate samples.

Peptides 8WH5, 7WH5 and 6WH5 had similar degradation profiles. For the three smallest peptides it was possible to visualize the band at 8 h (Figure 5C–E). For the first 4 h, the peptide amount in the collected aliquots was close to 90% (Table 2). Even at 6 and 8 h, the peptide amount present in these aliquots was close to or above 50% (Table 2), indicating a lower degradation rate over time for these peptides. Observing these results, we can say that peptides 8WH5, 7WH5 and 6WH5 were more resistant to proteolytic cleavage than WP11 and 0WHst53. The graph in Figure 6 shows this behavior, indicating that the degradation rate was slower for these peptides while more rapid for 0WHst5 and WP113.

**Figure 7:**
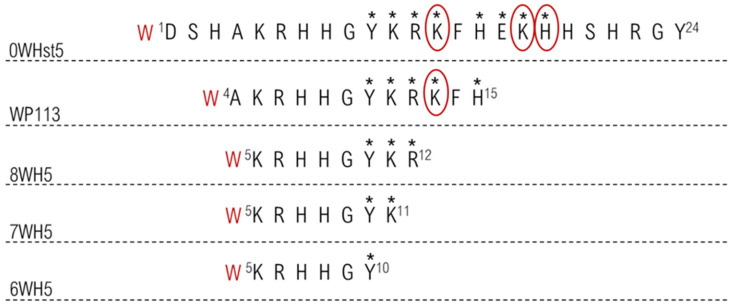
Proteolytic cleavage sites in the peptides target to salivary proteases. The stars indicate all primary cleavage sites and the circle indicates preferred primary cleavage sites.

These results may be related to the cleavage sites present in 0WHst5 and its analogues. The image in Figure 7 highlights all proteolytic cleavage sites found for Hst5 [29] and proposed cleavage sites present in the analogue peptides, since they conserved part of the original Hst5 sequence. As demonstrated, there were eight cleavage points (shown by *) in the 0WHst5 amino acid chain; seven of these were present in the functional domain (Y10–H15) [29]. Peptide WP113 had only 12 amino acids present in the functional domain of 0WHst5 (A4–H15). These sites were most likely attacked by proteolytic enzymes, explaining its rapid degradation and similar profile to the original peptide. Furthermore, the amino acid K13 was described as a preferential cleavage site for proteolytic enzymes in saliva [29], which makes 0WHst5 and WP113 a target for proteolytic cleavage and degradation.

The smaller peptides, 8WH5, 7WH5 and 6WH5 had fewer cleavage sites in their amino acid sequences and they did not possess any preferential cleavage sites. Thus, by removing the preferred sites from the amino acid sequence, these peptides were less susceptible to proteolytic degradation and were able to remain in saliva for a longer period of time. This result and the results obtained for the antifungal action for these peptides make the peptides 8WH5, 7WH5 and 6WH5 promising molecules for inhibiting *C. albicans* in the oral cavity.

### 3.5. High-Performance Liquid Chromatography (HPLC) Analysis

**Figure 8:**
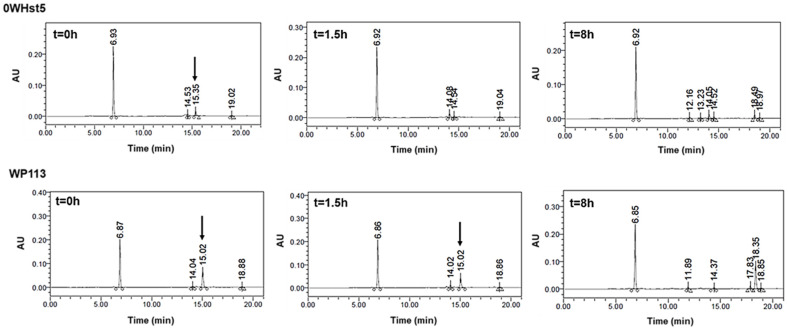
0WHst5 and WP113 degradation profile in human saliva. Aliquots of 100 µL were boiled and analyzed by HPLC after 0 h, 1.5 h and 8 h of incubation. Black arrows indicate intact 0WHst5 and WP113, retention time in 15.35 and 15.02 min, respectively.

The rapid degradation observed for 0WHst5 and WP113 during cationic-PAGE was also observed by HPLC. For 0WHst5, after 1.5 h of incubation in diluted WSS we were unable to observe the peak at 15.3 min characteristic for this peptide. It was still possible to see the peak for WP113 in 1.5 h, but it disappeared on the 8-hour chromatogram (Figure 8). Some reports are like those found here for 0WHst5, reporting rapid degradation for Hst5 after 1.5 h on WSS [29].

**Figure 9:**
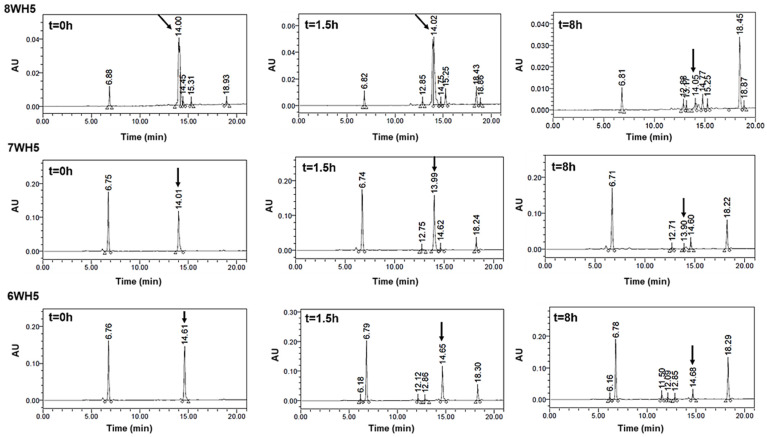
8WH5, 7WH5 and 6WH5 degradation profile in human saliva. Aliquots of 100 µL were boiled and analyzed by HPLC after 0 h, 1.5 h and 8 h of incubation. Black arrows indicate intact 8WH5, 7WH5 and 6WH5, retention time at 14.00, 13.99 and 14.61 min, respectively.

As can be seen in Figure 9, peaks representing peptides 8WH5, 7WH5 and 6WH5 were found in all the samples analyzed, indicating these peptides remained in the samples for 8 h. It is possible that 0WHst5 and WP113 peaks were below the HPLC detection limit after 8 h but 8WH5, 7WH5 and 6WH5 were still detectable at 8 h. Even though the initial amount of 8WH5, 7WH5 and 6WH5 was reduced over the time, it is important to emphasize that they were still present after more than 1.5 h. Thus, these peptides performed their antifungal action longer than 0WHst5 and WP113.

The design and synthesis of smaller peptides resulted in peptides less susceptible to the action of proteases present in human saliva. The strategy of decreasing the amino acid chain of 0WHst5 and removing the preferred sites for proteolytic cleavage (as shown in Figure 7), has proven successful. The result was smaller peptides which are faster and cheaper to synthesize. Furthermore, these peptides show improved antifungal activity and in the oral cavity.

### 3.6. LC-ESI-MS/MS Analysis

It is interesting to note that most of the fragments derived for 0WHst5 and WP113 conserved the amino acids necessary for the antifungal action, known by the functional domain of Hst5 (4A–H15) [19]. These data agree with Helmerhorst et al., who highlighted that the early degradation of Hst5 does not abolish its antifungal properties [28]. According to Table 3 and Table 4, for peptides WHst5 and WP1132, the fragments highlighted with (*) still had some vestiges of the functional domain of Hst5 in their sequence, thus suggesting that the antifungal action of these fragments would be active as described by Helmerhorst et al. [28]. Despite that, note that we found more fragments for WHst5 and WP113 than for 8WH5, 7WH5 and 6WH5, as can be seen in Table 3, Table 4, Table 5, Table 6 and Table 7. This could be due to the smaller peptides having fewer preferred sites for cleavage by salivary enzymes.

The peptides 8WH5, 7WH5 and 6WH5 were present in the samples with their complete amino acid sequence, as can be seen in the Table 5, Table 6 and Table 7 (his5 5/12; his5 5/11; his5 5/10 respectively). Only 7WH5 presented a fragment similar to that of its original sequence (Table 6; his5 6/11), but also quite similar to that of the 6WH5 peptide, with possible antifungal action. This result, together with what we observed via HPLC, shows that these peptides were less affected by salivary proteases, with less degradation and fragmentation (Table 6). Furthermore, these peptides showed good antifungal action, with inhibition even for the fluconazole-resistant strain, as shown in Figure 1. Thus, peptides 8WH5, 7WH5 and 6WH5 can be highlighted as the smallest fragments derived from Hst5 that still retained antifungal action. Smaller fragments that appeared in mass spec analyses, such as the sequences “G Y K R”, “F H E K H” and “A K R H”, did not have the amino acids corresponding to the functional domain of Hst5, suggesting to us that they would display negligible antifungal activity. Thus, using SPPS, it was possible to synthesize smaller peptides, which showed significant antifungal action within a relevant potency range compared to Hst5 and P-113, with much better proteolytic stability. Peptides 8WH5, 7WH5 and 6WH5 were less affected to salivary proteases, possibly because they do not possess in their amino acid chain the preferential cleavage sites for these enzymes. While the focus of this study is the proteolytic stability, ionic strength also affects AMP activity, especially in saliva. High salt levels can reduce microbial binding. Therefore, this strategy can also be used in future studies, for the in vivo use of these peptides.

## 4. Conclusions

In summary, our studies suggest that the three smallest engineered novel peptides (8WH5, 7WH5 and 6WH5) were more resistant to proteolytic cleavage, with improved antifungal activity compared to that of Hst5 and P-113. It is possible to suggest that the antifungal activity from Hst5 in human saliva is attributed to fragments generated by proteolytic cleavage in this environment. Together, these fragments can exert a very expressive antifungal action. The design and creation of new peptides, based on known peptide sequences such as Hst5, can provide the emergence of new structurally improved molecules. This is the case for 8WH5 which, in addition to showing inhibition in low concentrations against *C. albicans*, also demonstrated resistance against saliva-mediated proteolytic cleavage. Peptides 7WH5 and 6WH5 were even smaller than 8WH5 yet still preserved antifungal activity, and 7WH5 was effective against fluconazole-resistant *C. albicans*. Like 8WH5, these peptides were also shown to undergo less degradation and proteolytic fragmentation. Finally, our results demonstrate the potential of three new molecules with great potential to treat *C. albicans* infections in the oral cavity.

## Figures and Tables

**Table 1:** Amino acid sequence and physicochemical features of the synthetic peptides.

Peptide	Amino Acid Sequence	Net Charge *	MW (g mol^−1^)
0WHst5	**W** D S H A K R H H G Y K R K F H E K H H S H R G Y	+5.7	3225.50
WP113	**W** A K R H H G Y K R K F H	+5.3	1751.01
8WH5	**W** K R H H G Y K R	+4.2	1267.44
7WH5	**W** K R H H G Y K	+3.2	1111.26
6WH5	**W** K R H H G Y	+2.2	983.09

* Net charge obtained from theoretical pK values at physiological pH.

**Table 2:** Quantification bands for the peptides 0WHst5, WP113, 8WH5, 7WH5 and 6WH5 remaining after withdrawal of aliquots at times 0, 0.5, 1.5, 4, 6, 8, 24 and 48 h. Bands were analyzed and quantified using Image Lab 6.1 software (Bio-Rad Inc., Hercules, CA, USA).

Time (h)	0WHst5 (%)	WP113 (%)	8WH5 (%)	7WH5 (%)	6WH5 (%)
0	100.0 ± 0.0	100.0 ± 0.0	100.0 ± 0.0	100.0 ± 0.0	100.0 ± 0.0
0.5	91.0 ± 17.0	89.5 ± 7.3	99.9 ± 8.0	100.0 ± 4.5	98.8 ± 1.1
1.5	61.0 ± 19.8	56.3 ± 30.9	78.2 ± 13.9	76.8 ± 15.7	96.6 ± 4.6
4	-	7.23 ± 8.17	80.3 ± 13.0	86.2 ± 3.1	79.3 ± 9.8
6	-	-	62.0 ± 8.4	55.9 ± 5.8	52.4 ± 7.0
8	-	-	44.2 ± 0.9	49.6 ± 2.4	30.6 ± 4.0
24	-	-	8.6 ± 4.4	19.1 ± 7.5	16.5 ± 5.9
48	-	-	3.5 ± 0.8	6.9 ± 1.0	8.3 ± 6.2

**Table 3:** Amino acid sequences and properties of WHst5 fragments generated upon 48 h of incubation with diluted WSS.

0WHst5			
Name	Observed (M + H)+	Theoretical (M + H)+	Sequence
Histatin 5	-	3036.29	D S H A K R H H G Y K R K F H E K H H S H R G Y **
his_5_ 1/14	1766.93	1766.96	D S H A K R H H G Y K R K F *
his_5_ 7/24	2341.17	2341.55	H H G Y K R K F H E K H H S H R G Y *
his_5_ 8/24	2204.11	2204.41	H G Y K R K F H E K H H S H R G Y *
his_5_ 9/24	2067.05	2067.28	G Y K R K F H E K H H S H R G Y *
his_5_ 10/24	2010.03	2010.22	Y K R K F H E K H H S H R G *
his_5_ 11/24	1846.96	1847.05	K R K F H E K H H S H R G Y
his_5_ 12/24	1718.87	1718.88	R K F H E K H H S H R G Y
his_5_ 13/24	1562.77	1562.69	K F H E K H H S H R G Y
his_5_ 13/23	1399.70	1399.52	K F H E K H H S H R G
his_5_ 14/24	1434.67	1434.52	F H E K H H S H R G Y

(*) Fragments that maintained greater part of the functional domain. (**) Histatin 5 amino acid sequence with functional domain highlighted.

**Table 4:** Amino acid sequences and properties of WP113 fragments generated upon 48 h of incubation with diluted WSS.

WP113			
Name	Observed (M + H)+	Theoretical (M + H)+	Sequence
P113	-	1564.8	A K R H H G Y K R K F H **
his_5_ 1/21	2659.33	2659.88	D S H A K R H H G Y K R K F H E K H H S H *
his_5_ 1/15	1903.98	1904.13	D S H A K R H H G Y K R K F H *
his_5_ 1/14	1766.93	1766.96	D S H A K R H H G Y K R K F *
his_5_ 1/8	987.48	987.03	D S H A K R H H
his_5_ 2/15	1788.96	1789.02	S H A K R H H G Y K R K F H *
his_5_ 2/14	1651.90	1651.90	S H A K R H H G Y K R K F *
his_5_ 3/15	1701.93	1701.94	H A K R H H G Y K R K F H *
his_5_ 3/15	1564.87	1564.82	H A K R H H G Y K R K F *
his_5_ 3/8	785.42	784.87	H A K R H H
his_5_ 4/18	1959.06	1959.22	A K R H H G Y K R K F H E K H *
his_5_ 4/15	1564.87	1564.82	A K R H H G Y K R K F H *
his_5_ 4/14	1427.81	1427.66	A K R H H G Y K R K F *
his_5_ 5/24	2625.36	2625.91	K R H H G Y K R K F H E K H H S H R G Y *
his_5_ 5/15	1493.83	1493.72	K R H H G Y K R K F H *
his_5_ 5/13	1209.70	1209.41	K R H H G Y K R K *
his_5_ 6/21	2121.08	2121.33	R H H G Y K R K F H E K H H S H *
his_5_ 6/18	1759.93	1759.97	R H H G Y K R K F H E K H *
his_5_ 6/17	1622.87	1622.83	R H H G Y K R K F H E K *
his_5_ 6/15	1365.73	1365.55	R H H G Y K R K F H *
his_5_ 6/14	1228.68	1228.41	R H H G Y K R K F *
his_5_ 6/13	1081.61	1081.24	R H H G Y K R K *
his_5_ 7/18	1603.83	1603.79	H H G Y K R K F H E K H *
his_5_ 7/15	1209.63	1209.36	H H G Y K R K F H *
his_5_ 7/14	1072.57	1072.22	H H G Y K R K F
his_5_ 8/19	1603.83	1603.79	H G Y K R K F H E K H H *
his_5_ 8/15	1072.57	1072.22	H G Y K R K F H *
his_5_ 9/19	1466.77	1466.65	G Y K R K F H E K H H *
his_5_ 9/15	935.52	935.08	G Y K R K F H *
his_5_ 9/12	523.29	522.60	G Y K R
his_5_ 10/24	2010.03	2010.22	Y K R K F H E K H H S H R G Y
his_5_ 11/24	1846.96	1847.05	K R K F H E K H H S H R G Y
his_5_ 12/24	1718.87	1718.88	R K F H E K H H S H R G Y
his_5_ 12/22	1498.78	1498.65	R K F H E K H H S H R
his_5_ 13/24	1562.77	1562.69	K F H E K H H S H R G Y
his_5_ 14/24	1434.67	1434.52	F H E K H H S H R G Y
his_5_ 14/18	697.34	696.76	F H E K H
his_5_ 15/21	911.4	910.94	H E K H H S H

(*) Fragments that maintained greater part of the functional domain. (**) P113 amino acid sequence with functional domain highlighted.

**Table 5:** Amino acid sequences and properties of 8WH5 fragments generated upon 48 h of incubation with diluted WSS.

8WH5			
Name	Observed (M + H)+	Theoretical (M + H)+	Sequence
8WH5	-	1081.24	K R H H G Y K R **
his_5_ 1/12	1491.76	1491.62	D S H A K R H H G Y K R *
his_5_ 3/12	1289.70	1289.45	H A K R H H G Y K R *
his_5_ 4/7	511.30	510.59	A K R H
his_5_ 5/13	1209.70	1209.41	K R H H G Y K R K *
his_5_ 5/12	1081.61	1081.24	K R H H G Y K R *
his_5_ 6/19	1896.99	1897.11	R H H G Y K R K F H E K H H

(*) Fragments that maintained greater part of the 8WH5 peptide. (**) 8WH5 amino acid sequence.

**Table 6:** Amino acid sequences and properties of 7WH5 fragments generated upon 48 h of incubation with diluted WSS.

7WH5			
Name	Observed (M + H)+	Theoretical (M + H)+	Sequence
7WH5	-	925.05	K R H H G Y K **
his_5_ 1/14	1766.93	1766.99	D S H A K R H H G Y K R K F *
his_5_ 5/13	1209.70	1209.42	K R H H G Y K R K *
his_5_ 5/11	925.51	925.06	K R H H G Y K *
his_5_ 6/11	797.41	796.88	R H H G Y K *
his_5_ 7/13	925.51	925.05	H H G Y K R K
his_5_ 12/17	844.47	843.97	R K F H E K

(*) Fragments that maintained greater part of the 7WH5 peptide. (**) 7WH5 amino acid sequence.

**Table 7:** Amino acid sequences and properties of 6WH5 fragments generated upon 48 h of incubation with diluted WSS.

6WH5			
Name	Observed (M + H)+	Theoretical (M + H)+	Sequence
6WH5	-	796.88	K R H H G Y **
his_5_ 5/10	797.41	796.88	K R H H G Y *
his_5_ 10/24	2010.03	2010.22	Y K R K F H E K H H S H R G Y

(*) Fragments that maintained greater part of the 6WH5 peptide. (**) 6WH5 amino acid sequence.

## Data Availability

The original contributions presented in this study are included in the article. Further inquiries can be directed to the corresponding authors.

## Abbreviations

The following abbreviations are used in this manuscript:

Hst5	Histatin 5
AFP	Antifungal Peptide
SPPS	Solid Phase Peptide Synthesis
AMP	Antimicrobial Peptides
MIC	Minimum Inhibitory Concentration
SDA	Sabouraud Dextrose Agar
SDB	Sabouraud Dextrose Broth
DIC	Diisopropyl-carbodiimide
HoBt	N-hydroxybenzotriazole
DMF	Dimethylformamide
DCM	Methylene chloride
TFA	Trifluoroacetic acid
EDT	1,2-Ethanedithiol
TIS	Triisopropylsilane
ACN	Acetonitrile
OD	Optical density
CLSI	Clinical and Laboratory Standards Institute
FLU	Fluconazole
WS	Whole saliva
WSS	Whole saliva supernatant
